# Ability of metformin to deplete NAD+ contributes to cancer cell susceptibility to metformin cytotoxicity and is dependent on NAMPT expression

**DOI:** 10.3389/fonc.2023.1225220

**Published:** 2023-07-31

**Authors:** Yongxian Zhuang, Allison B. Haugrud, Meg A. Schaefer, Shanta M. Messerli, W. Keith Miskimins

**Affiliations:** ^1^ Cancer Biology and Immunotherapies, Sanford Research, Sioux Falls, SD, United States; ^2^ Sanford Program for Undergraduate Research (SPUR) Program, Sanford Research, Sioux Falls, SD, United States

**Keywords:** metformin, nicotinamide adenine dinucleotide (NAD+), nicotinamide phosphoribosyltransferase (NAMPT), tumorigenesis, breast cancer

## Abstract

**Background:**

Nicotinamide adenine dinucleotide (NAD+) is vital for not only energy metabolism but also signaling pathways. A major source of NAD+ depletion is the activation of poly (ADP-ribose) polymerase (PARP) in response to DNA damage. We have previously demonstrated that metformin can cause both caspase-dependent cell death and PARP-dependent cell death in the MCF7 breast cancer cells but not in the MDA-MB-231 (231) breast cancer cells while in high-glucose media. We hypothesize that depletion of NAD+ in MCF7 cells via activation of PARP contributes to the cell death caused by metformin. Nicotinamide phosphoribosyltransferase (NAMPT), a key rate-limiting step in converting nicotinamide (vitamin B3) into NAD+, is essential for regenerating NAD+ for normal cellular processes. Evidence shows that overexpression of NAMPT is associated with tumorigenesis. We hypothesize that NAMPT expression may determine the extent to which cancer cells are sensitive to metformin.

**Results:**

In this study, we found that metformin significantly decreases NAD+ levels over time, and that this could be delayed by PARP inhibitors. Pretreatment with NAD+ in MCF7 cells also prevents cell death and the enlargement of mitochondria and protects mitochondria from losing membrane potential caused by metformin. This leads to MCF7 cell resistance to metformin cytotoxicity in a manner similar to 231 cells. By studying the differences in NAD+ regulation in these two breast cancer cell lines, we demonstrate that NAMPT is expressed at higher levels in 231 cells than in MCF7 cells. When NAMPT is genetically repressed in 231 cells, they become much more sensitive to metformin-induced cell death. Conversely, overexpressing NAMPT in HEK-293 (293) cells causes the cells to be more resistant to metformin’s growth inhibitory effects. The addition of a NAMPT activator also decreased the sensitivity of MCF7 cells to metformin, while the NAMPT activator, P7C3, protects against metformin**-**induced cytotoxicity.

**Conclusions:**

Depletion of cellular NAD+ is a key aspect of sensitivity of cancer cells to the cytotoxic effects of metformin. NAMPT plays a key role in maintaining sufficient levels of NAD+, and cells that express elevated levels of NAMPT are resistant to killing by metformin.

## Introduction

Metformin, a commonly prescribed anti-type 2 diabetes drug, has gained attention due to its effects on cancer cell growth and survival (1–10) and possible effects on reducing cancer risk in type 2 diabetes patients who take metformin (11–13). This has been suggested to be related to its effects on inhibition of cell growth (1, 3, 4, 6, 14, 15) and induction of cell death (2, 10, 16–18). Our previous study demonstrates that metformin causes both caspase-dependent and poly (ADP-ribose) polymerase (PARP)-dependent cell death (10). The activation of poly (ADP-ribose) polymerase-1 (PARP-1), a nuclear enzyme that catalyzes the synthesis of long, branching (ADP-ribose) polymers (PAR) from nicotinamide adenine dinucleotide (NAD+), can transfer multiple ADP-ribose moieties from NAD+ to nuclear proteins and to PARP itself (19–23). This process is also expected to deplete the NAD+ reservoir.

NAD+ is required for multiple reactions by regulating numerous enzymes including dehydrogenases, poly(ADP-ribose) polymerases, Sir2 family proteins (sirtuins), mono (ADP ribosyl) transferases, and ADP-ribosyl cyclases (24). By regulating the aforementioned enzymes, NAD+ levels intercede in cell cycle progression, DNA repair, metabolic regulation, and circadian rhythms, which are important in the pathogenesis in various diseases and the functionality of various cells. Several studies have shown that maintaining NAD+ level in the cell can profoundly decrease cell death (25–27). These studies have fundamentally changed our understanding about NAD+, suggesting novel paradigms about the metabolism and biological activities of NAD+. Consumption of NAD+ severely compromises ATP synthesis, whereas synthesis of NAD+ requires ATP during glycolysis and oxidative phosphorylation (28, 29). Therefore, activation of PARP may lead to an energy deficit and contribute to cell death caused by metformin.

Understanding how cancer cells maintain and replenish NAD+ levels is essential in understanding if depleting NAD+ reserves through metformin treatment will work. Nicotinamide phosphoribosyltransferase (NAMPT) is a rate-limiting enzyme in the regeneration of NAD+ from nicotinamide. Mammalian cells can use other sources such as nicotinic acid or tryptophan to produce NAD+, even though using nicotinamide through NAMPT is the most efficient and predominant manner of regenerating NAD+ (30–32). Although crucial to normal systemic functionality, NAMPT has recently been associated with tumorigenesis (33), and its increased expression is associated with the pathogenesis or various cancers (34–36). NAMPT may prove to be a key factor in determining the resiliency of cancers to cancer therapies, which induce metabolic stress or DNA damage.

In this study, we explore metformin’s effects on NAD+ levels, if PARP plays a role in NAD+ depletion, and how NAD+ levels affect metformin cytotoxicity. Since NAMPT plays an essential role in NAD+ replenishment, we explore how NAMPT levels can affect cancer susceptibility to metformin. We hypothesize that metformin-induced depletion of NAD augments the cytotoxicity following metformin exposure.

## Materials and methods

### Chemicals and cell culture

Metformin (1, 1-dimethylbiguanide, #D5035, Sigma, St. Louis, USA), PARP inhibitor II (INH2BP, #407850, Calbiochem, USA), MCF7, 293T, and 231-MDA-MB (231) cells were purchased from ATCC (Manassas, USA) and maintained in Dulbecco’s modified Eagle’s medium (DMEM) with 10% fetal bovine serum supplemented with 100 U/ml penicillin and 100 µg/ml streptomycin in a humidified incubator with 5% CO_2_. Upon receiving the cell lines, cells were immediately cultured and expanded to prepare frozen ampule stocks. Cells were passaged for no more than 2–3 months before establishing new cultures from the early passage frozen ampules. Cell lines were routinely checked for mycoplasma contamination and were verified to be mycoplasma free by IDEXX RADIL Laboratories.

### Trypan blue exclusive assay

For the MCF7 and 293T experiments, cells were plated into 35-mm dishes. For the 231 experiments, cells were plated in a 24-well plate. The following day, cells were treated as indicated. After incubation for the indicated time, cells were trypsinized and stained using 0.2% trypan blue. Trypan blue-positive cells and -negative cells were determined using a hemacytometer.

### Phase microscopy

Cells were treated as indicated and pictures were taken at 200× magnification using a Zeiss Axiovert 35 light microscope equipped with a Nikon digital camera.

### Confocal microscopy

MCF7 cells that stably transfected with pAcGFP-Mito Vector were plated on the cover glass in 35-mm dishes and treated as indicated. Cells were washed in 1× PBS and fixed in 4% paraformaldehyde for 15 min. Finally, slides were washed in 1× PBS three times and mounted using Vectorshield medium containing DAPI. Slides were observed using an Olympus FV1000 confocal microscope. PAcGFP1-Mito Vector was purchased from Clontech, USA.

### Flow cytometry

MCF7 cells were treated as indicated in the figure. Then, cells were incubated with 100 nM TMRE for 10 min, and cells were washed in 1× PBS once and trypsinized. All flow cytometry measurements were done using an Accuri C6 flow cytometer.

### Western blot

Cells were plated on 35-mm dishes. After treatment for the indicated time period, live cells were harvested and lysed by the addition of sodium dodecyl sulfate (SDS) sample buffer [2.5 mM Tris–HCl (pH 6.8), 2.5% SDS, 100 mM dithiothreitol, 10% glycerol, and 0.025% bromophenol blue]. Equal amounts of protein were separated on a 4%–20% Mini-PROTEAN^®^ TGX™ Gel (Bio-Rad Laboratories). Proteins were transferred to Trans-Blot^®^ Turbo™ RTA Mini PVDF with a Bio-Rad Trans-blot apparatus using a transfer buffer [48 mM Tris–HCl, 39 mM glycine]. The membranes were immersed in 5% non-fat dry milk in Tris-buffered saline containing Tween20 (TBS-T) [10 mM Tris–HCl (pH 7.5), 150 mM NaCl, and 0.1% Tween-20] with an NAMPT antibody (Raybiotech cat # RR08-0003) for either 3 h at room temperature or 4°C overnight. After thoroughly washing with TBS-T, an appropriate secondary antibody conjugated to horseradish peroxidase (HRP) was applied. The membrane was again washed, and proteins were then detected using GE Healthcare Amersham™ ECL™ Prime Western Blotting Detection Reagent imaged on a BioSpectrum Imaging System (UVP, LLC).

In the NAD+ assay for the 231 experiments, cells were grown at 75% confluence in 60-mm dishes. For the 293T experiments, 300,000 cells were plated in each well in a six-well plate. The following day, 231 cells were treated with 2.5 mM glucose complete media while 293T cells received 10 mM glucose complete media and appropriate dishes were treated with 8 mM metformin. After 24 h of treatment, cells were trypsinized and counted via a hemacytometer. Cells were then rinsed with 1× PBS and vortexed in NAD extraction buffer included in the EnzyChrom™ NAD/NADH Assay Kit (BioAssay Systems) and kit directions were followed from this point. The 231 experiments used 300,000 cells/sample while the 293T experiments used 500,000 cells/sample.

For MCF7 experiments, MCF7 cells were grown to 75% confluence in 60-mm tissue culture dishes, with 500, 000 cells plated per dish. After plating for 24 h, the following drugs were added: Metformin (8 mM), P7C3 (5 µM), and Metformin (8 mM) combined with P7C3 (5 µM). NAD was obtained using the EnzyChrom NAD/NADH (E2ND-100) kit as instructed by the vendor BioAssay.

### ATP assay

A total of 150,000 231 cells were plated in each well of a 12-well plate. The following day, 2.5 mM glucose complete media was added along with indicated treatments. After 24 h of treatment, cells were trypsinized and counted via a hemacytometer. A total of 100,000 cells were placed in a tube and rinsed with 1× PBS. ATP Determination Kit (Life Technologies) was used to determine ATP content.

### Cytotoxicity assay

A total of 1,000 MCF7 cells were plated in a 96-well plate, and concentrations of P7C3 (Selleckchem) ranging from 0 to 5 µM in the presence and absence of metformin (10 mM) were added 24 h after plating. After drug administration for 48 h, a cytotoxicity assay was performed using Sytox Green (Invitrogen). After adding Sytox Green for 15 min, 0.6% Triton X was added to determine the total number of cells.

### xCELLigence data

After a background reading was completed on E-plates, 20,000 231 NAMPT knockdown stable cells were plated per well. Cells were allowed to plate at room temperature for 20 min before placing plates in the xCELLigence Real-Time Cell Analyzer (RTCA) (Acea Biosciences. Inc.). The following day, fresh media and treatments were added as indicated. Cell index, which is a dimensionless measurement of electrical impedance from cells that reflects cell number, cell morphology, and adhesion, was measured.

### Construction of stable cell lines

MDA-MB-231 cells were retrovirally transduced using a NAMPT shRNA-mir (clone ID RLGH-GU42575, Transomic Technologies). Puromycin (0.5 µg/ml) was used to select out stables. Once colonies were established, cloning rings were used to isolate colonies. Western blotting was used to determine the level of NAMPT protein expression.

### Overexpression of NAMPT

HEK**-**293 cells were plated at 300,000 cells/well in a six-well plate in triplicate. The following day, NAMPT mRNA (GeneCopeia Cat # EX-A1275-M02) or empty vector (GeneCopeia Cat # EX-NEG-M02) were transfected using Dreamfect (OZ Biosciences). Forty-eight hours later, the NAD assay was performed as described above.

### Statistical analysis

Comparison of two groups was done using unpaired *t*-test in GraphPad Prism software (La Jolla, CA). *p*-values less than or equal to 0.05 were considered to have significance.

## Results

**Figure 1:**
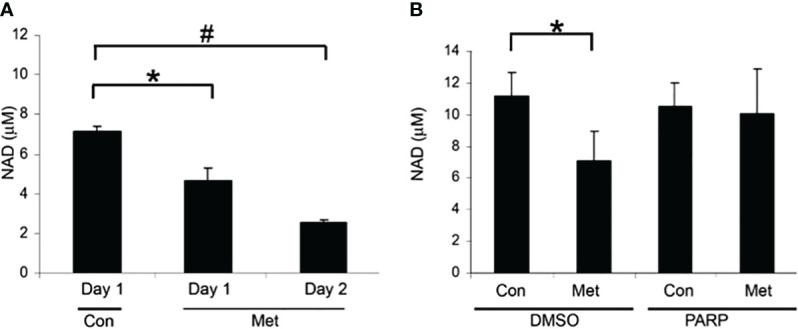
Metformin decreased NAD+ levels after 1 day of treatment and can partially be blocked by PARP inhibitors. **(A)** NAD+ levels of MCF7 cells that were treated with 8 mM metformin as indicated. **(B)** NAD+ levels of MCF7 cells that were pretreated with a PARP inhibitor (10 μM) for 1 day and treated subsequently with metformin (8 mM) for 1 or 2 additional days in the presence of the PARP inhibitor. The same number of cells was used for each sample in the NAD assay (true for all subsequent NAD+ assays). (* and # indicate significant difference between groups with *p* < 0.05.) Lanes labeled Con represent cells not treated with metformin and lanes labeled Met denote those treated with metformin. Lanes labeled PARP indicate cells treated with a PARP inhibitor and DMSO indicates control samples not treated with PARP inhibitor.

Our previous data have shown that metformin causes cell death in MCF7 cells via caspase-dependent and PARP-dependent cell death (10). Since NAD+ is used in PARP activation [19-23], we looked at the NAD+ level in association with PARP-dependent cell death. In **Figure 1A**, we evaluated the level of NAD+ after 1 and 2 days of metformin treatment. After 1 day of treatment, NAD+ levels significantly decreased and further decreased on day 2. To verify the link between PARP and NAD+ levels, a PARP inhibitor was utilized. In **Figure 1B**, MCF7 cells were pretreated with the PARP inhibitor for 1 day and then treated with or without metformin for an additional 2 days. The PARP inhibitor pretreatment prevented the reduction of NAD+ caused by metformin treatment. This suggests that the depletion of NAD+ caused by metformin is partially due to the activation of PARP.

**Figure 2:**
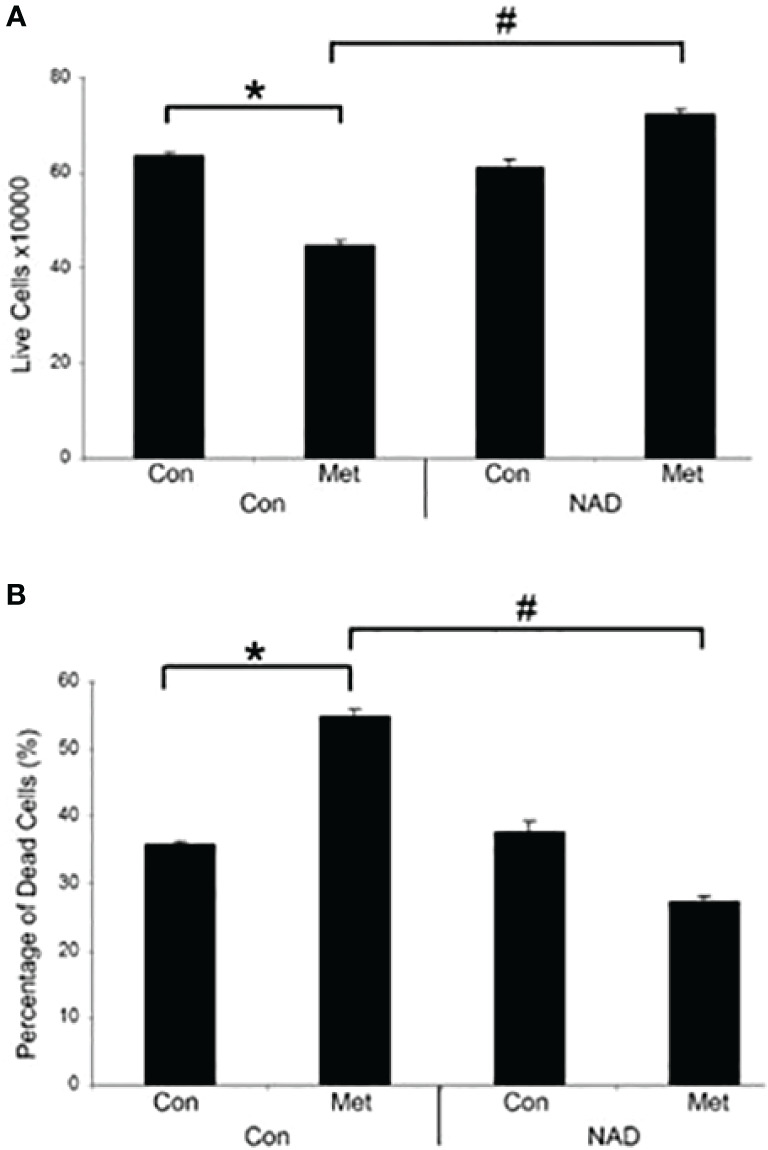
Metformin decreases live cells and induces cell death *in vitro*, which can be prevented by the addition of exogenous NAD+. MCF7 cells were pretreated with NAD+ (1 mM) for 3 days and subsequently treated with or without metformin (8 mM) for 2 days. Dead and live cell numbers were obtained using the trypan blue exclusion assay. (* and # indicate significant difference between groups with *p* < 0.05.).

Our previous data have shown that PARP inhibition could partially prevent the cell death caused by metformin (10). This correlates with the prevention of the decrease of NAD+ shown in **Figure 1**, so we assessed whether the addition of exogenous NAD+ to the media could delay the cell death caused by metformin. MCF7 cells were pretreated with NAD+ for 3 days and then treated with or without metformin for 2 days. As seen in **Figure 2**, NAD+ pretreatment increased the number of live cells (**Figure 2A**) and prevented the cell death (**Figure 2B**) caused by metformin, which further confirms that the cell death is partially caused by depletion of NAD+ by metformin.

**Figure 3:**
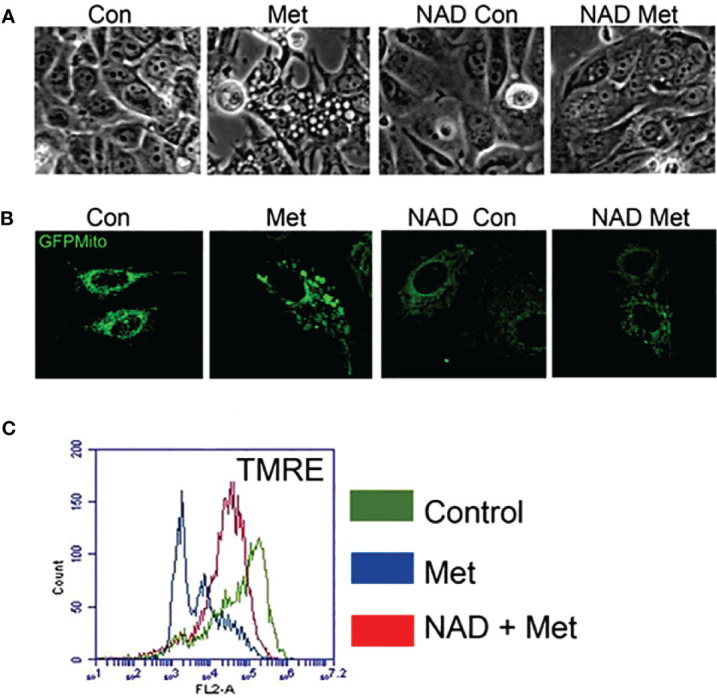
Pretreatment of MCF7 cells with NAD+ prevents the enlargement of mitochondria and protects mitochondria from losing membrane potential caused by metformin. MCF7 cells were pretreated with NAD+ (1 mM) for 3 days and then treated with or without metformin for 2 days. **(A)** Phase contrast image of MCF7 cells. **(B)** MCF7 stable cells expressing pAcGFP1-Mito Vector were visualized using confocal microscopy to see the morphology of the mitochondria. **(C)** Cells were stained using TMRE. Flow cytometry was used to detect the change of mitochondrial membrane potential.

As shown in our previous study, metformin can cause enlargement of mitochondria, which could be prevented by a PARP inhibitor (10). We next studied whether pretreating with exogenous NAD+ could prevent mitochondrial enlargement. MCF7 stable cells expressing pAcGFP-Mito Vector were pretreated with NAD+ for 3 days, then treated with or without metformin for 2 days. **Figure 3A** displays phase contrast pictures showing an accumulation of large, clear vesicles present in the metformin-treated cells while the pretreatment of NAD+ with metformin lacks these vesicles. Confocal images shown in **Figure 3B** confirm that the large vesicles are enlarged mitochondria, which was also demonstrated in our previous paper. The NAD+ pretreatment prevented the enlargement of mitochondria caused by metformin. Furthermore, membrane potential was investigated since NAD+ has been documented to be involved in the membrane potential maintenance (37). MCF7 cells were pretreated with NAD+ for 3 days and were subsequently treated with or without metformin for 2 days. NAD+ pretreatment could partially reverse metformin’s effects on mitochondrial membrane potential as seen in **Figure 3C**. NAD+ protects the mitochondrial membrane potential from metformin, allowing the mitochondria to maintain morphology leading to the delay of cell death. This finding further elucidates the mechanism of metformin function in causing cell death in MCF7 breast cancer cells.

**Figure 4:**
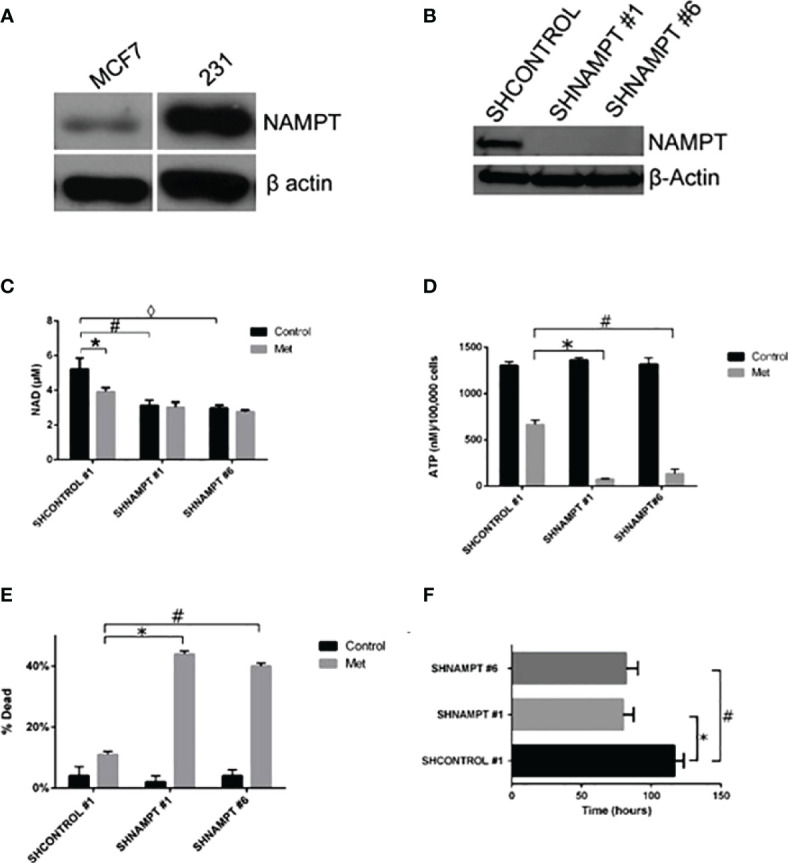
231 cells overexpress NAMPT compared to MCF7 cells. When NAMPT is knocked down in 231 cells, NAD+ and ATP levels are depleted following metformin treatment, inducing metformin sensitivity. **(A)** Western blot showing that metformin-resistant 231 cells have higher levels of NAMPT compared to metformin-sensitive MCF7 cells. **(B)** Western blot showing 231 stable cells expressing shNAMPT have low NAMPT expression. **(C)** NAMPT knockdown leads to lower NAD+ levels after 1 day of treatment with 8 mM metformin (*p = 0.029, #*p* = 0.0069, ◊*p* = 0.004). **(D)** ATP depletion was also seen with the same treatments (*p = 0.000029, #*p* = 0.00018). **(E)** Enhanced cell death in shNAMPT stables following metformin treatment shown in a Trypan Blue exclusion assay (*p = 0.0000024, #*p* = 0.0000037). **(F)** Depiction of the time that the cell index reached less than 1 after 8 mM metformin treatment (*p = 0.0003, #*p* = 0.0008).

Since NAD+ levels are important in determining the fate of a cancer cell to surviving a therapy, we examined if the protein NAMPT, the rate-limiting enzyme in NAD biosynthesis, played a role in a cancer cell line resistance to metformin treatment. In our previous study, we found that the breast cancer cell line MDA-MB-231 (231) cells were resistant to metformin’s apoptotic effects in culture media containing 25 mM glucose. Of note is that in another study of ours, 231 cells were metformin sensitive when incubated in a lower glucose level media (38). In **Figure 4A**, NAMPT protein levels were examined in MCF7 and 231 cells via Western blot. 231 cells had much higher levels of NAMPT, which may partially account for the metformin resistance in 231 cells in high-glucose media while MCF7 cells are sensitive.

To elucidate the role that NAMPT plays in protecting 231 cells from metformin-induced apoptosis, we created stable knockdowns of NAMPT and confirmed expression of NAMPT by Western blot in **Figure 4B**. To verify that the NAMPT knockdown causes a decrease in NAD+, a NAD+ assay was performed as shown in **Figure 4C**. Indeed, there was a significant decrease of NAD+ levels comparing the SHCONTROL and SHNAMPT cell lines in the control treatment. When treated with metformin, the SHCONTROL line had a significant decrease in NAD+ compared to control treatment. Interestingly, when the SHNAMPT cells were treated with metformin, they were not able to reach an even lower level of NAD+.

Since NAD+ is not only involved in PARP activation but also a key factor in maintaining metabolism, an ATP assay was performed as shown in **Figure 4D**. The SHCONTROL cells had a large drop in ATP levels when treated with metformin. However, an even more striking significant drop in ATP was found between the SHCONTROL metformin-treated cells and the SHNAMPT metformin-treated cells, indicating that maintaining NAD+ levels is also important for the maintenance of ATP.

We have shown that NAMPT expression is important in maintaining NAD+ and ATP levels when cells are under metabolic stress, such as following metformin-induced ROS activity [Haugrad, 2014]. We hypothesized that NAMPT overexpression would be involved in determining cancer cell sensitivity to metformin. In **Figure 4E**, a trypan blue exclusion assay was performed, which displayed the significant increase in cell death in SHNAMPT metformin-treated cells compared to SHCONTROL metformin-treated cells after 1 day. To further confirm this result, an xCELLigence assay was utilized to track the time the cell index reached less than one while cells were treated with metformin in low glucose (2.5 mM glucose). As shown in **Figure 4F**, the two 231 SHNAMPT stables reached a cell index of less than 1 with an estimated 37 h sooner than the 231 SHCONTROL cells, which confirms that the status of NAMPT may determine the rate at which the 231 cells die from metformin and low-glucose treatment.

**Figure 5:**
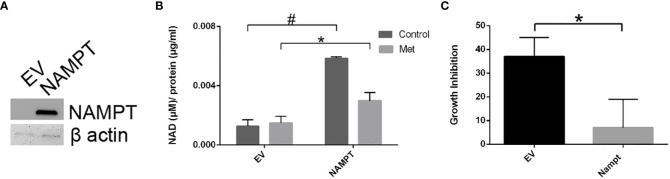
293T cell NAMPT overexpression results in higher NAD+ levels and lower growth inhibition in the presence of metformin. **(A)** Western blot analysis following transfection of NAMPT mRNA or Empty Vector (EV) in 293T cells. **(B)** Following NAMPT mRNA transfection, 293T cells were treated for 1 day, counted via trypan exclusion assay (*p = 0.023, #*p* = 0.00060). **(C)** Growth inhibition analysis via Trypan Blue assay of 293T mRNA transfection followed by metformin treatment (*p = 0.023).

**Figure 6:**
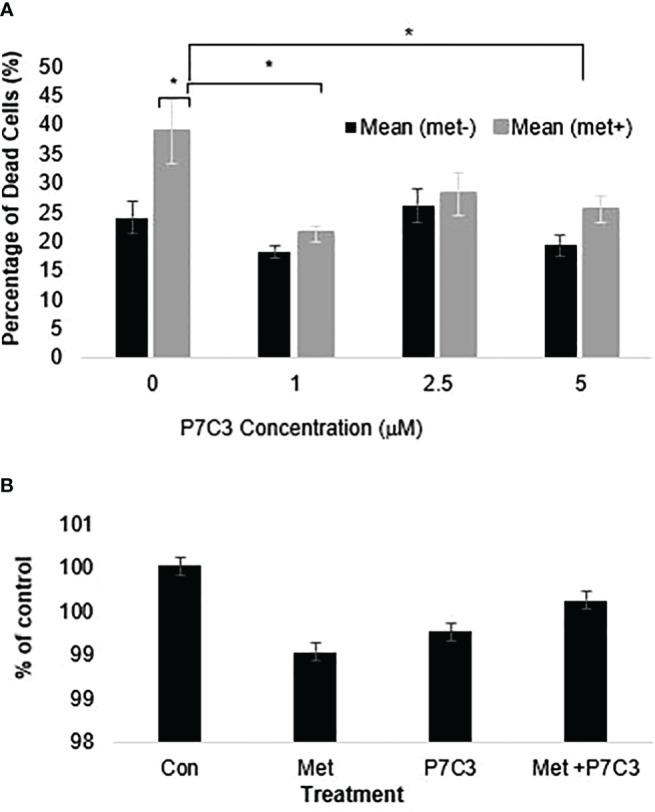
**(A)** P7C3 protects against metformin-induced cytotoxicity and reduces NAD. Sytox Green assays indicate that P7C3 significantly protected against metformin-induced cytotoxicity at 1 and 5 μM P7C3, with error bars denoting standard deviation, according to an unpaired *t*-test with *p* < 0.05 (*). **(B)** NAD assays conducted after 48 h indicate that metformin (8 mM) reduces NAD while 5 mM P7C3 protects against metformin-induced NAD reduction. NAD levels were increased when P7C3 was combined with metformin as compared to metformin alone.

To further support the importance of NAMPT and NAD levels in cells, we examined how overexpression of NAMPT affected NAD+ levels and cytotoxicity. We used 293T cells to overexpress NAMPT as shown in Western blot analysis in **Figure 5A**. Overexpressing NAMPT in 293T cells led to statistically significant higher NAD levels not only in the control-treated cells but also in the metformin-treated cells (**Figure 5B**). **Figure 5C** shows that the higher levels of NAMPT significantly prevented growth inhibition when 293T cells were treated with metformin. Similarly, activation of NAMPT with P7C3 in MCF7 cells significantly reduced metformin-induced cytotoxicity at concentrations of 1 and 5 μM P7C3 (**Figure 6A**). Increased NAD levels were observed when P7C3 was combined with metformin as compared to metformin alone (**Figure 6B**). The NAMPT activator P7C3 protects against metformin-induced cytotoxicity, suggesting that increased NAD+ levels may hinder metformin-induced cytotoxicity.

## Discussion

Our present findings support the importance of NAD+ in sensitivity of cancer cells to metformin cytotoxicity. In our previous study (10), we showed that metformin induces cell death in MCF7 cells in a caspase-dependent and PARP-dependent manner. As supported by other literature (39) and by other studies of ours (38, 40), metformin inhibits complex I of the electron transport chain. This inhibition leads to an accumulation of reactive oxygen species (ROS) causing DNA damage. In response to the DNA damage, PARP is activated and uses NAD+ to create PAR to start DNA repair. While DNA repair is advantageous to the cancer cell, overactivation of PARP leads to NAD+ depletion. NAD+ is not only involved in DNA repair but also necessary for glycolysis and oxidative phosphorylation. Metformin, by inducing PARP overactivation, may cause a “metabolic catastrophe” as supported in the depletion of ATP in **Figure 4D** in cells with low NAD+ levels (38).

As demonstrated in our previous work, metformin induced ROS production in mitochondria as measured by flow cytometry using the dye Mitosox in MCF7 cells (40). Our unpublished data illustrate that 231 SHNAMPT stables experienced ROS levels similar to those of SHCONTROL cells in response to metformin using the same assay. Even though metformin induced similar oxidative stress in 231 SHCONTROL and SHNAMPT stable cell lines, 231 SHNAMPT cells had significant increased sensitivity to metformin cytotoxicity, which is due to their already depleted NAD+ available to the cells to maintain metabolism while combating DNA damage. Our studies on both MCF7 and 231 cells further highlight the importance of NAMPT in maintaining cellular NAD+ levels and in the ability of cancer cells to maintain an energy balance to avoid cancer therapy cytotoxicity. Metformin or other therapies inducing DNA damage may cause metabolic catastrophes through NAD+ shortage in some tumors but may not be as effective in other tumors depending on the levels of NAMPT expression.

Even though intensive studies have been done to understand the mechanisms of metformin on cancer cell growth inhibition and cell death, it has been shown that there is quite a bit of variation in response to metformin in different cancer cells (2, 16). By further understanding the differences between cancer cells, it may shed light on further understanding mechanisms of metformin and provide important information in the application of metformin for cancer prevention and treatment

The group of Isakovic has shown that metformin caused massive induction of caspase-dependent apoptosis associated with c-Jun N-terminal kinase (JNK) activation, mitochondrial depolarization, and oxidative stress in confluent C6 cultures, and metformin-triggered apoptosis was completely prevented by a mitochondrial permeability transition blocker (cyclosporin A) (2). However, our unpublished data indicate that cyclosporin A could not block the cell death caused by metformin in MCF7 cells. This suggests that even though metformin can cause apoptotic cell death in different cell types, the mechanisms of cell death may not be the same and may depend on the response of mitochondria to metformin treatment in various cell types. As shown in **Figure 3A**, MCF7 cells respond to metformin with the enlargement of mitochondria when in high-glucose media, which can be prevented with the addition of NAD+ or a PARP inhibitor (10). This also brings the possible link between the morphological changes of mitochondria and cell death. Furthermore, while metformin-sensitive MCF7 cells have drastic mitochondrial morphology changes, metformin-resistant 231 cells that have higher NAMPT expression do not, leading us to propose that mitochondrial dynamics and metabolic phenotype are different in these two cell lines while in high-glucose media. However, as mentioned before, we have shown an enhanced sensitivity to metformin in low-glucose media in both MCF7 and 231 cells while MCF10A cells were left unaffected (38), showing promise for the specificity of this treatment to be on tumor cells. Interestingly, mitochondrial changes were not seen in the MCF7 cells in low-glucose/metformin treatment. This is due to an enhanced metabolic catastrophe that does not allow time for mitochondrial morphology changes.

Future studies on mitochondrial dynamics and proteins involved will further aid in understanding why metformin is more effective in some cell lines than others. Such studies will provide essential information concerning the potential use of metformin as an anti-cancer drug and also in understanding the drug mode of action in the treatment of type 2 diabetes.

NAMPT, the critical rate-limiting enzyme of the NAD+ salvage pathway, has recently become an intriguing protein for a target in creating new drug therapies or for being used as a biomarker. NAMPT levels are often significantly elevated in a number of tumor types (33, 36), are associated with advanced tumor progression (35), and can be induced by hypoxia, which is another marker of poor prognosis (34). Here, we demonstrate that the level of NAMPT expression determines the sensitivity of cells to metformin treatment, which makes NAMPT an attractive target for drug development. NAMPT inhibitors such as FK866 (also known as APO866) have shown promise in *in vitro* and *in vivo* studies. For example, *in vitro*, FK866 induces cell death in both chemotherapy-sensitive and -resistant small cell lung cancer cell lines (40). In chronic lymphocytic leukemia, FK866 has been identified as a possible targeted therapeutic in high-risk patients, and three clinical trials have been completed showing that FK866 was well tolerated (41). These drugs could be used as cancer sensitizers to treatments that induce NAD+ depletion such as a low-glucose diet or metformin.

Our overall model is that metformin inhibits Complex I of the electron transport chain (NADH:ubiquinone oxidoreductase), leading to an increase in superoxide and accumulation of ROS. Metformin-mediated increases in cellular ROS causes DNA damage, which promotes an increase in PARP activity. This is supported by studies that show that when PARP is inhibited, there is a decrease in metformin-induced cell death. Since PARP uses NAD+ as the substrate to synthesize poly(ADP-ribose), metformin-induced PARP activity leads to NAD+ depletion. Therefore, treating with exogenous NAD + or increasing NAMPT activity reduces metformin-induced cell death, while knocking down NAMPT levels enhances metformin-induced cell death.

Overall, this study highlights the role that NAD+ availability plays in not only metformin treatment, but also other potential treatments. NAMPT expression may considerably alter how cancer cells may react to different treatments. If further large-scale clinical trials confirm the antitumor effects of metformin, this drug may become an alternative cancer adjuvant therapy in conjunction with standard-of-care chemotherapeutics.

## Data availability statement

The datasets presented in this article are not readily available because There are no datasets which require filing as this paper does not involve any genomic or transcriptomic data. Requests to access the datasets should be directed to Keith.Miskimins@sanfordhealth.org.

## Author contributions

YZ, AH, and SM wrote the manuscript. YZ, AH, MS, and SMM performed the laboratory experiments. YZ, AH, SMM, and WM edited the manuscript. WM provided conceptualization. WM and SM provided funding for work. All authors contributed to the article and approved the submitted version.
